# Effects on heart pumping function when using foam and gauze for negative pressure wound therapy of sternotomy wounds

**DOI:** 10.1186/1749-8090-6-5

**Published:** 2011-01-13

**Authors:** Malin Malmsjö, Sandra Lindstedt, Richard Ingemansson

**Affiliations:** 1 Department of Ophthalmology, Lund University Hospital, Lund, Sweden; 2 Department of Cardiothoracic Surgery, Lund University Hospital, Lund, Sweden

## Abstract

**Background:**

Negative pressure wound therapy (NPWT) has remarkable effects on the healing of poststernotomy mediastinitis. Foam is presently the material of choice for NPWT in this indication. There is now increasing interest in using gauze, as this has proven successful in the treatment of peripheral wounds. It is important to determine the effects of NPWT using gauze on heart pumping function before it can be used for deep sternotomy wounds. The aim was to examine the effects of NPWT when using gauze and foam on the heart pumping function during the treatment of a sternotomy wound.

**Methods:**

Eight pigs underwent median sternotomy followed by NPWT at -40, -70, -120 and -160 mmHg, using foam or gauze. The heart frequency, cardiac output, mean systemic arterial pressure, mean pulmonary artery pressure, central venous pressure and left atrial pressure were recorded.

**Results:**

Cardiac output was not affected by NPWT using gauze or foam. Heart frequency decreased during NPWT when using foam, but not gauze. Treatment with foam also lowered the central venous pressure and the left atrial pressure, while gauze had no such effects. Mean systemic arterial pressure, mean pulmonary artery pressure and systemic vascular resistance were not affected by NPWT. Similar haemodynamic effects were observed at all levels of negative pressure studied.

**Conclusions:**

NPWT using foam results in decreased heart frequency and lower right and left atrial filling pressures. The use of gauze in NPWT did not affect the haemodynamic parameters studied. Gauze may thus provide an alternative to foam for NPWT of sternotomy wounds.

## Background

Cardiac surgery is complicated by poststernotomy mediastinitis in 1-5% of all procedures [1]; a complication that is life-threatening [2]. The reported early mortality using conventional therapy is between 8 and 25% [3,4]. In 1999, Obdeijn and colleagues described the treatment of poststernotomy mediastinitis using vacuum-assisted closure [5], now called negative pressure wound therapy (NPWT). The technique entails the application of negative pressure to a sealed wound. NPWT has remarkable effects on the healing of poststernotomy mediastinitis, and has reduced the rate of mortality considerably [6]. The organs in the mediastinum are haemodynamically crucial, and both vulnerable bypass grafts and heart function should be taken into consideration when performing NPWT after cardiac surgery.

NPWT is known to affect the heart pumping function, although the results from different studies on the subject are not consistent [7-11]. Using sonometry, Conquest and colleagues showed that subatmospheric pressure decreased the left ventricular volume and cardiac output in pigs by approximately 30% [7], but that this could be prevented by rotating a rectus muscle flap over the mediastinal wound. In a subsequent study by Petzina et al., using magnetic resonance imaging, NPWT of sternotomy wounds in pigs was shown to decrease the cardiac output to a lesser extent, 13% [10]. In studies by Sjögren et al. and Steigelman et al., using thermodilution, cardiac output was found not to be affected by negative pressure [8,11].

Foam is presently the material of choice when applying NPWT to deep sternotomy wounds. However, interest in the use of gauze has increased [12,13], as this has proven successful in the treatment of other kinds of peripheral wounds [14]. The advantages with gauze include its conformability and ease of application to large and irregular wounds [18]. It has been suggested that gauze under negative pressure can tamponade superficial bleedings in sternotomy wounds [19]. Furthermore, there are no reported problems with ingrowth of granulation tissue into gauze in NPWT [20]. Before gauze is used for NPWT of sternotomy wounds, it is important to determine the effects on heart pumping function. Hitherto, the effect of NPWT on the pumping function of the heart has only been examined using foam [7-10]. The aim of the present study was therefore to compare the haemodynamic effects of NPWT using gauze and foam. Eight pigs underwent median sternotomy and the wounds were treated with NPWT at negative pressures of -40, -70, -120 or -160 mmHg, using foam or gauze. Haemodynamic parameters, including heart frequency, cardiac output, mean systemic arterial pressure, mean pulmonary artery pressure, central venous pressure and left atrial pressure, were recorded.

## Methods

### Animals

A porcine sternotomy wound model was used. Eight domestic landrace pigs with a mean body weight of 70 kg were fasted overnight with free access to water. The study was approved by the Ethics Committee for Animal Research, Lund University, Sweden. The investigation complied with the "Guide for the Care and Use of Laboratory Animals" as recommended by the U.S. National Institutes of Health and published by the National Academies Press (1996).

### Anaesthesia and surgery

Premedication was performed with an intramuscular injection of xylazine (Rompun^® ^vet. 20 mg/ml; Bayer AG, Leverkusen, Germany; 2 mg/kg) mixed with ketamine (Ketaminol^® ^vet. 100 mg/ml; Farmaceutici Gellini S.p.A, Aprilia, Italy; 20 mg/kg). Before surgery, a tracheotomy was performed and an endo-tracheal tube was inserted. Anaesthesia was maintained with a continuous infusion of ketamine (Ketaminol^® ^vet. 50 mg/ml; 0.4-.6 mg/kg/h). Complete neuromuscular blockade was achieved with a continuous infusion of pancuronium bromide (Pavulon; N.V. Organon, OSS, the Netherlands; 0.3-0.5 mg/kg/h). Fluid loss was compensated for by continuous infusion of Ringer's acetate at a rate of 300 ml/kg/h. Mechanical ventilation was established with a Siemens-Elema ventilator (Servo Ventilator 300, Siemens, Solna, Sweden) in the volume-controlled mode (65% nitrous oxide, 35% oxygen). Ventilatory settings were identical for all animals (respiratory rate: 15 breaths/min; minute ventilation: 8 l/min). A positive end-expiratory pressure of 5 cmH_2_O was applied. A Foley catheter was inserted into the urinary bladder through a suprapubic cystostomy. Upon completion of the experiments, the animals were euthanized with a lethal dose (60 mmol) of intravenous potassium chloride.

### Wound preparation for NPWT

A midline sternotomy was performed. Three layers of paraffin gauze Jelonet^® ^(Smith & Nephew, Hull, UK) were placed over the anterior surface of the heart to protect it from the sternal edges. The wound was filled with saline-soaked AMD gauze (RENASYS-G, St Petersberg, FL), or one of two kinds of foam: VAC foam size 18 × 12.5 × 3.3 cm (VAC^® ^black GranuFoam^®^, KCI, San Antonio, TX), or RENASYS-F foam, 20 × 12.5 × 3 cm (RENASYS-F, St Petersberg, FL). One layer of foam or two rolls of gauze were placed between the sternal edges. A second layer of foam or two rolls of gauze were placed over the first layer, between the soft tissue wound edges, and secured to the surrounding skin. Two drains were inserted into the wound filler. One drain was placed between two layers of wound filler (foam or gauze) and the other was placed at the top of the wound filler The wound was sealed with a transparent adhesive drape and connected to a vacuum source (RENASYS-EZ, Smith & Nephew St Petersburg, FL). The vacuum source was set to deliver negative continuous pressures of -40, -70, -120 and -160 mmHg.

### Haemodynamic assessment

The mean systemic arterial pressure was monitored via a catheter in the left carotid artery. The mean pulmonary artery pressure was monitored via a catheter in the pulmonary artery. Double-lumen central venous catheters were inserted into the left external jugular vein and the left atrium to record the central venous pressure and left atrial pressure, respectively. A flow probe (CardioMed TraCe System, Medistim, Norway) was placed around the pulmonary artery to record the cardiac output. The catheter was connected to a cardiac output monitor (Oximetrix 3, Abbot Laboratories, North Chicago, IL, USA). The haemodynamic data were collected in a data acquisition system (PowerLab, AD Instruments Ltd., Castle Hill, Australia). Systemic vascular resistance and pulmonary vascular resistance were calculated from the above parameters.

After surgical preparation, the animal was allowed to stabilize for one hour. Baseline measurements of the above mentioned haemodynamic parameters were then recorded before applying NPWT. Negative pressures of -40, -70, -120 or -160 mmHg were applied, and the effects on the haemodynamic parameters were recorded after 2½ and 5 min of NPWT. The negative pressures were applied in a random order, with intervals of 10 min without any pressure.

### Limitations

The study was performed using an acute sternotomy wound model in pigs where the organs in the thoracic cavity can move freely. In patient s with mediastinitis there are typically adhesions around the heart that may restrict movement. In addition, following a short period of NPWT, the mediastinum becomes fixed and rigid with the development of granulation tissue and fibrous ingrowth. The hemodynamic effects of NPWT may differ between an acute porcine sternotomy wound model and patients with mediastinitis.

### Calculations and statistics

Calculations and statistical analysis were performed using GraphPad 5.0 software (San Diego, CA, USA). The effect of NPWT on each haemodynamic parameter was calculated as a percent of the baseline value. Statistical analysis was performed using the Mann-Whitney test. Significance was defined as *P *< 0.05 (*), *P *< 0.01 (**), *P *< 0.001 (***) and *P *> 0.05 (not significant, n.s.). Values are presented as means ± the standard error on the mean (S.E.M.) unless otherwise stated.

## Results

**Figure 1:**
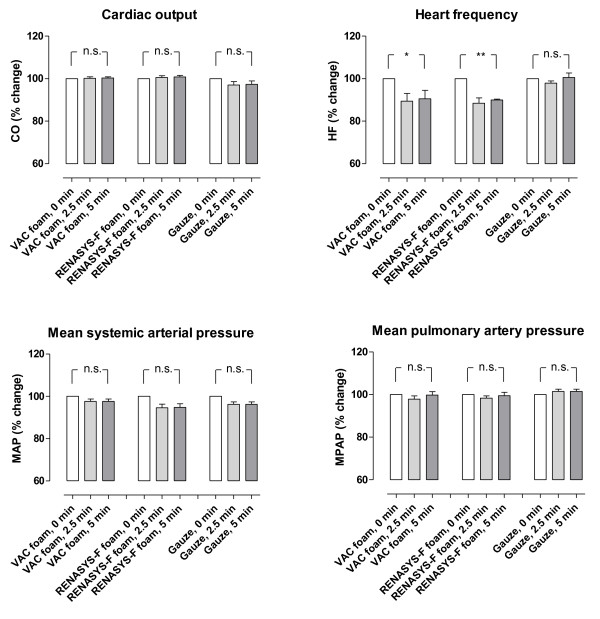
**Cardiac output, heart frequency, mean systemic arterial pressure and mean pulmonary artery pressure before NPWT is applied (0 min) and 2½ and 5 min after a negative pressure of -120 mmHg was applied**. Measurements were made during NPWT using gauze or two different types of foam. The results are shown as means ± the standard error on the mean of eight experiments. Each of the 8 pigs was subjected to NPWT using all 3 different dressings (n = 8). Statistical analysis was performed using the Mann-Whitney test. Significance was defined as *P *< 0.05 (*), *P *< 0.01 (**), *P *< 0.001 (***) and *P *> 0.05 (not significant, n.s.). Before negative pressure was applied, baseline heart frequency was 114 ± 1 bpm, cardiac output was 3.0 ± 0.1 L/min, mean systemic arterial pressure was 85 ± 3 mmHg and mean pulmonary artery pressure was 17.5 ± 0.5 mmHg. Note that heart frequency decreases during NPWT using foam, but not gauze. Results were similar at the other negative pressures studied (not shown).

**Figure 2:**
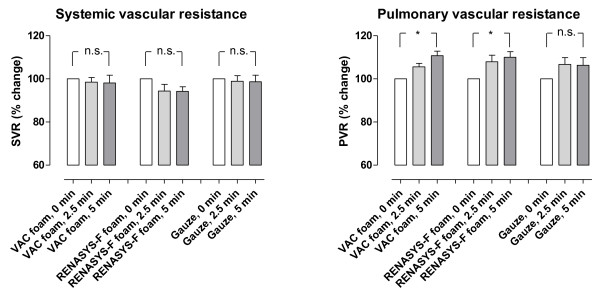
**Change in systemic vascular resistance and pulmonary vascular resistance upon the application of NPWT at -120 mmHg, using foam and gauze (n = 8)**. Before negative pressure was applied, baseline systemic vascular resistance was 1957 ± 43 dynes*sec/cm^5 ^and pulmonary vascular resistance was 172 ± 3 dynes*sec/cm^5^. Results were similar at the other negative pressures studied (not shown).

**Figure 3:**
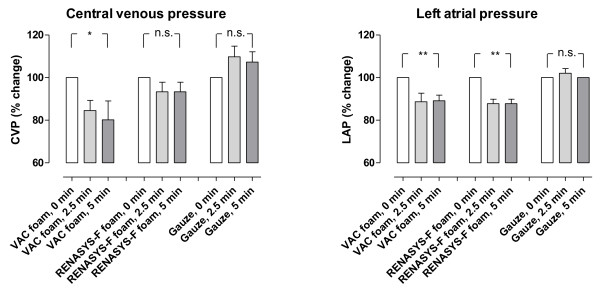
**Change in central venous pressure and left atrial pressure upon the application of NPWT at -120 mmHg, using foam and gauze (n = 8)**. Before negative pressure was applied, baseline left atrial pressure was 6.3 ± 0.2 mmHg and central venous pressure was 4.8 ± 0.1 mmHg. Results were similar at the other negative pressures studied (not shown).

Cardiac output was not affected by NPWT when using gauze or foam (p = n.s., Figure 1). Heart frequency was decreased (-9%, p < 0.05) when NPWT was applied using foam, but not when using gauze (p = n.s., Figure 1). Neither the mean arterial pressure nor the mean pulmonary artery pressure was affected by NPWT (p = n.s., Figure 1). NPWT did not alter the systemic vascular resistance. The pulmonary vascular resistance increased, as a result of calculating it by a formula with the left atrial pressure (Figure 2). Negative pressure treatment with foam lowered the central venous pressure (-20%, p < 0.05) and the left atrial pressure (-11%, p < 0.01), while gauze had no such effects (p = n.s., Figure 3).

The effects of NPWT on the haemodynamic parameters were immediate, and the recordings after 2½ and 5 min of therapy were similar (Figures 1, 2 and 3). Comparable haemodynamic effects were observed at all levels of negative pressure studied (-40, -70, -120 and -160 mmHg). There was no significant difference in the haemodynamic effects when using the two different types of foam.

## Discussion

### Cardiac output

The present findings regarding cardiac output agree largely with those in previous studies, although the results in these studies vary somewhat. Mohktari et al. reported that the haemodynamics in pigs with a sternotomy wound treated with NPWT at -75 mmHg was unaltered, using an ultraflow probe to measure the flow through the pulmonary artery [9]. Furthermore, in a study by Steigelman et al., using theromdilution, cardiac output was unaltered by a negative pressure of -125 mmHg [11]. In a study by Sjögren et al., using thermodilution, the cardiac output was found to be slightly increased when a sternotomy wound in pigs was treated with a negative pressure of -75, while other negative pressures (-50, -100, -125, -150 and -175 mmHg) had no effect [8]. In a study by Petzina et al., cardiac output was reported to be slightly reduced during NPWT (-75, -125 and -175 mmHg) of sternotomy wounds in pigs, when measured using magnetic resonance imaging [10].

The effect of NPWT on cardiac output is especially important since many patients with deep sternal wound infections have impaired cardiac function and heart failure due to ischaemic heart disease. Their ability to compensate for a decrease in cardiac output during NPWT may thus be limited. It has been suggested that haemodynamic parameters should be carefully monitored in patients undergoing sternal NPWT [7]. However, when considering the data from the present study and other reports [8-10], it appears that NPWT has no major effects on cardiac output, and invasive monitoring may therefore be unnecessary.

### Heart rate

The results of the present study show that the heart frequency decreased when NPWT was applied using foam. Similar results have been found in an experimental study on NPWT of a sternotomy wound using foam, demonstrating a tendency towards decreased heart rated upon the application of negative pressures of -75, -125 and -175 mmHg [10]. Interestingly, the present study shows that gauze has no such effects. The reason for this cannot be deduced from the present study, but may be due to differences in the mechanical effects by the two materials on the heart [17]. The decrease in heart rate when using foam is only slight (~20%) and the heart pumping function may only be affected when compensatory mechanisms are compromised as a result of advanced heart failure.

### Left and right atrial filling pressure

**Figure 4:**
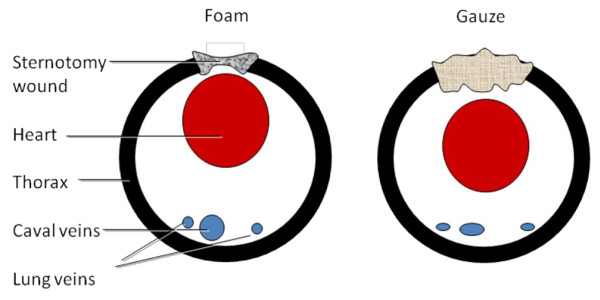
**Illustrations of the cross-section of the thoracic cavity showing an open sternotomy, the heart, and caval and lung veins**. Suction in a sternotomy wound by NPWT results in the heart being displaced outwards, towards the sternum. There is a greater reduction in the volume of the foam than the gauze, as the latter is a denser material. The heart may therefore be drawn outwards to a greater extent when using foam than when using gauze. NPWT using foam may relieve tension on the caval and lung veins at the back of the thoracic cavity, thereby decreasing the filling pressure in the right and left atria. This may be haemodynamically beneficial, as the load on the heart is reduced.

This study shows that the use of foam in NPWT lowers the central venous pressure and the left atrial pressure. We suggest that this is also the result of differences in the mechanical effects on the heart and large intrathoracic vessels when using foam and gauze. Suction in a sternotomy wound results in the heart being displaced outwards, towards the sternum [17]. Foam allows a greater volume reduction than gauze, as the latter is a denser material. The heart may therefore be drawn outwards to a greater extent when using foam than when using gauze. NPWT using foam may relieve tension on the caval and lung veins in the bottom of the thoracic cavity, thereby decreasing the filling pressure in the right and left atria. This is haemodynamically beneficial as the load on the heart is reduced. The suggested mechanism is illustrated in Figure 4.

### Clinical implications

The results of this and previous studies indicate that careful consideration should be given to the choice of material used in NPWT, as both foam and gauze may have advantages and disadvantages [21]. Both result in the formation of granulation tissue; foam leads to the growth of a thick layer of tissue, which may penetrate the foam, making it difficult to remove after NPWT. Gauze leads to the growth of thin, dense granulation tissue without ingrowth, commonly referred to by physicians as high-quality tissue. NPWT using gauze has proven to be successful in the treatment of other kinds of wounds [14].

## Conclusions

This study was performed to compare the effects of NPWT of a sternotomy wound using foam and gauze on heart pumping function. Foam is presently the material of choice for NPWT in cardiac surgery. NPWT using foam results in a slight decrease in heart frequency and lower right and left atrial filling pressures. NPWT using gauze does not affect the heart pumping function. Gauze may provide an alternative to foam for the treatment of sternotomy wounds using NPWT.

## Competing interest

The authors declare that they have no competing interests.

## Authors' contributions

MM; designed study and wrote the manuscript. SL; collected and analysed the data. RI; designed the study. All authors have read and approved the final manuscript.
